# Genital Lichen Sclerosus—The Role of the Vulvar Microbiome

**DOI:** 10.3390/medicina61091632

**Published:** 2025-09-09

**Authors:** Sandra Jerkovic Gulin, Olivia Almlöf, Oliver Seifert, Georgios Kravvas, Filippa Lundin, Malin Bergman Jungeström, Jan Söderman

**Affiliations:** 1 Department of Dermatology and Venereology, Ryhov County Hospital, 551 85 Jönköping, Sweden; oliver.seifert@liu.se; 2 Division of Cell Biology, Department of Biomedical and Clinical Sciences, Faculty of Medicine and Health Sciences, Linköping University, 581 83 Linköping, Sweden; 3 Faculty of Medicine and Health Sciences, Linköping University, 581 83 Linköping, Sweden; 4 Department of Dermatology, University College London Hospitals NHS Foundation Trust, London NW1 2BU, UK; georgios.kravvas@nhs.net; 5 Department of Medicine, University College London, London WC1E 6JF, UK; 6 Department of Clinical Microbiology, Department of Biomedical and Clinical Sciences, Faculty of Medicine and Health Sciences, Linköping University, 581 83 Linköping, Sweden; 7 Department of Biomedical and Clinical Sciences, Faculty of Medicine and Health Sciences, Linköping University, 581 83 Linköping, Sweden; 8 Laboratory Medicine, Ryhov County Hospital, 551 85 Jönköping, Sweden

**Keywords:** lichen sclerosus, vulvar microbiome, vulvar lichen sclerosus

## Abstract

*Background and Objectives:* Lichen sclerosus (LSc) is a chronic inflammatory and sclerosing condition that primarily affects the genital region. In women, vulvar lichen sclerosus presents with a wide range of clinical features, including pruritus, erythema, burning, pain, dysuria, architectural distortion, scarring, and the formation of ivory-white atrophic plaques. While the precise etiology of LSc remains unclear, increasing evidence indicates that prolonged exposure to urine and occlusion may play a central role in its pathogenesis. Additionally, the role of the genital microbiome has garnered increasing attention, with dysbiosis—an imbalance in microbial communities—emerging as a potential contributing factor. Prior investigations have reported an increased abundance of *Gardnerella* in women with LSc, but findings regarding *Streptococcus* and *Lactobacillus* have been inconsistent. This study aimed to investigate the relationship between the vulvar microbiome and LSc. *Materials and Methods:* Forty-seven women with LSc and seven healthy controls were recruited from the Division of Dermatology, Ryhov Hospital, Jönköping. Vulvar swabs were obtained from affected skin areas. The 16S rRNA regions were amplified and sequenced using the Illumina MiSeq system. Differential abundance analyses were conducted using four regression methods, and genera were considered significant only if identified by at least two methods. *Results:* Compared to controls, LSc patients showed reduced abundance of *Lactobacillus*, *Finegoldia*, *Lawsonella*, *Stapylococcus*, *Cutibacterium*, and an unidentified genus belonging to the *Actinomycetaceae* family. In contrast, *Porphyromonas* and *Ezekiella*, were significantly increased. Beta diversity analysis revealed significant differences in microbiome composition between groups (*p* = 0.022). *Conclusions:* The vulvar microbiome of women with LSc differs significantly from that of healthy controls. These findings support a possible role of microbial dysbiosis in LSc pathogenesis, highlighting the need for further research into microbiome-targeted interventions.

## 1. Introduction

Lichen sclerosus (LSc) is a chronic inflammatory and sclerosing disease predominantly affecting the genital area, although extragenital manifestations on sites such as the trunk, breasts, or upper limbs may also occur, underscoring its broader clinical spectrum [1,2]. Histologically, LSc is characterized by basal cell vacuolization, epithelial atrophy, homogenization of the papillary dermis, and paradoxical hyperkeratosis. These features may overlap with morphea, where dermal atrophy predominates; however, the two entities can also coexist and represent distinct histopathological patterns [3]. Symptoms in women include pruritus, pain, and sometimes dysuria. Common signs include scarring, architectural effacement, white atrophic plaques, hyperkeratosis, and erosions [1,4]. Although the exact pathogenesis of genital LSc remains elusive, growing evidence suggests that prolonged exposure to urine and occlusion may play a central role [5,6,7]. Additional factors, such as autoimmunity, hormonal influences, genetic predisposition, and mechanical trauma, have been implicated, though none have been conclusively proven. Previous studies have suggested a possible association between Borrelia afzelii infection and lichen sclerosus, as Åsbrink et al. demonstrated in a cohort of Swedish patients with acrodermatitis chronica atrophicans, where lichen sclerosus–like lesions were occasionally observed [8]. To date, no infectious agent has been definitively identified [1].

Topical corticosteroids remain the cornerstone of treatment, often used in conjunction with barrier preparations. In men with refractory disease, circumcision offers an effective therapeutic option [9]. Early, effective treatment is essential, as it significantly alleviates symptoms, improves quality of life, and reduces the risk of sexual and urological morbidity and progression to malignancy [2,10]. The disease also imposes a substantial psychosocial burden. In women, particularly those postmenopause, LSc negatively impacts sexual function and sleep quality [11].

More recently, attention has turned to the potential role of the genital microbiome in LSc. Studies investigating the vulvar and vaginal microbiota in LSc have produced mixed results. Pagan et al. reported altered microbial diversity in women with LSc, including reduced *Prevotella* and increased *Papillomaviridae* [12]. Similarly, Pyle et al. observed decreased alpha diversity at the vulvar sites of women with LSc [13]. Conflicting findings have been reported regarding *Lactobacillus*, with some studies noting dominance [13,14], others highlighting elevated *Lactobacillus iners* [14] and still others reporting reductions in *Lactobacillus*, *Gardnerella*, and *Atopobium*, accompanied by increases in *Anaerococcus*, *Ezakiella*, and related genera [15,16]. The most frequently observed genera in LSc include *Lactobacillus, Gardnerella, Anaerococcus*, and *Staphylococcus* [16,17]. However, similar dominant taxa have also been found in healthy controls, with differences more often reflected in microbial richness or the clustering of less common genera. Notably, microbial composition also appears to vary by anatomical sampling site [17]. In male patients, microbial shifts have also been observed; for instance, the abundance of *Fusobacterium* species was found to be higher in men with LSc compared to healthy controls [18].

These heterogeneous findings, often derived from small cohorts and varied methodologies, underscore the need for larger, rigorously designed studies to clarify the role of microbial dysbiosis in LSc pathogenesis [14,15,16]. This study aims to characterize the vulvar microbiome in women with LSc compared to healthy controls using a standardized approach. We hypothesize that the microbial composition differs significantly between groups, which may offer novel insights into disease mechanisms and inform future diagnostic and therapeutic strategies.

## 2. Methods

### 2.1. Study Design and Population

This prospective case–control study was conducted at the Division of Dermatology, Ryhov Hospital, Jönköping. Forty-seven adult female patients with clinically diagnosed LSc and seven healthy female controls were recruited to the study. The diagnoses were made by specialist dermatologists with experience in female genital dermatoses.

Only adult female participants were eligible for inclusion in the study. Exclusion criteria included current pregnancy, active malignancy (excluding extra-genital basal cell or squamous cell carcinoma), active use of systemic or topical antibiotics, corticosteroids, or immunomodulatory agents applied to the genital area, and inability to understand Swedish or provide informed consent. All participants provided written informed consent for inclusion in the study and were enrolled with minimal disruption to their routine clinical care.

Samples were collected from the mons pubis, labia minora, and labia majora using flocked swabs (eNAT system, Copan Diagnostics, Copan Diagnostics, Murrieta, CA, USA). Topical treatments were discontinued at least one week before sampling.

### 2.2. Sample Preparation and 16S rRNA Variable Region Sequencing

Samples were pretreated with Proteinas K, and DNA extracted using the EZ1 Tissue kit v.2.0 and Biorobot EZ1 or EZ2 (Qiagen, Hilden, Germany). 16S rRNA variable regions were amplified, and sequencing libraries were produced from the DNA samples using the QIAseq 16S/ITS screening panels and index kit (Qiagen). Samples were sequenced and demultiplexed using the MiSeq system (Illumina, San Diego, CA, USA), with paired-end sequencing (2 × 300) using V3 sequencing chemistry.

Fastq sequences were demultiplexed based on the 16S variable region, and amplification primers were removed using the QIAseq 16S/ITS Demultiplexer tool in the Microbial Genomics Module of the CLC Genomic Workbench (Qiagen Digital Insights, Aarhus, Denmark). The Data QC and OTU Clustering tool of the Microbial Genomics Module was used to analyze the v3v4 regions of the 16S rRNA, quality trim the sequences, cluster them into operational taxonomic units (OTUs), and remove chimeric sequences. Based on a 99% similarity threshold, the samples were clustered using the “non-redundant” Silva database SSU Ref NR 99 (v138.1). By applying a 99% identity criterion, highly similar sequences were removed. The OTUs corresponding to, e.g., chloroplast and human DNA such as mitochondria, were filtered out, leaving 5650 OTUs aggregated at the genus level (450 genera).

### 2.3. Statistical Analysis

The count data for each sample and bacterial genera, as well as the metadata, were imported into R software (version 4.4.0; CRAN: https://cran.r-project.org/) for data processing and analysis. The packages used were from either CRAN or Bioconductor version 3.20 (https://bioconductor.org/), with the specific package names and versions detailed below. After filtering the count data to remove the bacterial genera (OUTs) based on prevalence (<0.25) and frequency (<0.005%) [19], 64 genera remained for further analysis. Different methods of normalization and transformation were applied depending on the analysis performed.

The effect of differences in sequencing depth on the alpha diversity was investigated through rarefaction curves (vegan package version 2.6-10), with richness used as the measurement metric. Samples with fewer than 10,000 counts were excluded. Differences in the beta diversity were examined with the vegan package using the Bray–Curtis dissimilarity measure and permutational multivariate analysis of variance (PERMANOVA). They were further visualized with principal coordinates analysis (PCoA) using the cmdscale function from R stats package (R version 4.3.3).

Regression analyses were performed using four different methods, in order to avoid bias by a single method, to examine the differential abundance of genera between the vulvar microbiome in women with LSc and the vulvar microbiome in healthy control subjects. Two different methods in the MaAslin2 package (version 1.20.0) were applied, i.e., either total sum scaling (TSS) as a normalization method, in conjunction with a log transformation, or the centered log-ratio (CLR) transformation. Additionally, both the DESeq2 (package version 1.46.0) and the limma voom/LmFit functions (combined in the voomLmFit function of the edgeR package version 4.4.2) were used, and to handle zero-inflated data, the poscounts estimator was used for DESeq2 and the TMMwsp method for the voomLmFit function. To be considered significant, a result had to be confirmed by at least two of the methods. *p* values were corrected using the Benjamini–Hochberg procedure, and a false discovery rate (FDR) of 0.25 was applied as a cutoff. Overlapping results were visualized using a Venn diagram (ggVennDiagram package version 1.5.2).

Abundance data (centered log-ratio transformed count data) were visualized using principal coordinates (PCA; prcomp function from R stats package) and heatmaps (pheatmap package version 1.0.12).

## 3. Ethics

The ethical approval for this study was obtained on 15 November 2021: dnr. 2021-05590-01 and issued by the Swedish Ethical Review Authority.

## 4. Results

### 4.1. Deseq2

The genera *Finegoldia*, *Dialister*, *Lactobacillus,* and *Lawsonella* were found in decreased amounts in the women with LSc compared to the healthy controls (adjusted *p* < 0.25). *Helcoccucus*, *Ezakiella*, *Negativicoccus*, *Parvimonas*, *Actinotignum*, *Actinobaculum*, *Atopobium*, *Fenollaria*, *Porphyromonas*, *Bacteroides*, *Gardnerella*, *Subdoligranulum*, and an unverified genus possibly belonging to the order *Eubacteriales* were found in significantly increased abundance in the women with LSc compared to the healthy controls using Deseq2 (adjusted *p* < 0.25).

### 4.2. MaAslin2 CLR

The genera *Lactobacillus*, *Staphylococcus*, *Finegoldia*, *Lawsonella, Cutibacterium*, and an unknown genus in the family *Actinomycetaceae* were found in decreased abundance in the women with LSc (adjusted *p* < 0.25). *Porphyromonas* and *Ezakiella* were found in increased abundance (adjusted *p* < 0.25).

### 4.3. Limma

*Lactobacillus*, *Finegoldia*, *Lawsonella*, *Staphylococcus*, *Cutibacterium*, and an unknown genus in the family *Actinomycetaceae* were found in decreased abundance in the women with LSc compared to the healthy controls. No genera were found to be significantly increased.

**Figure 1:**
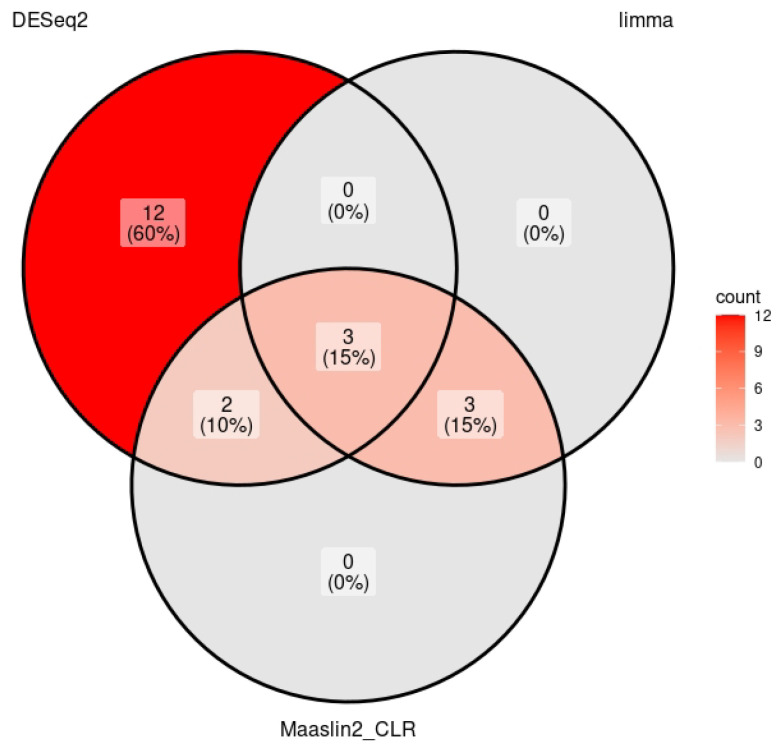
Number of genera found to significantly differ in abundance when comparing women with LSc to healthy controls, using the methods DESeq2, limma, and MaAslin2 CLR. Depicted using a Venn diagram.

Three of the genera were found in statistically significant decreased abundance in the women with LSc compared to the healthy controls, in all three analyses. These were *Lactobacillus*, *Finegoldia*, and *Lawsonella*. The genera *Staphylococcus*, *Cutibacterium*, and an unknown genus belonging to the family *Actinomycetaceae* were found in decreased abundance in women with LSc with Masslin2 CLR and limma but not with Deseq2. The genera *Porphyromonas* and *Ezekiella* were found in increased abundance after analysis using MaAslin2 CLR and Deseq2, but not limma (Figure 1). All eight of these genera are summarized in Table 1.

### 4.4. Beta Diversity

Significant association between sample type and the microbiome was found when analyzing the beta diversity of the two groups with the 64 genera remaining after filtering for frequency and prevalence (*p* = 0.022).

**Figure 2:**
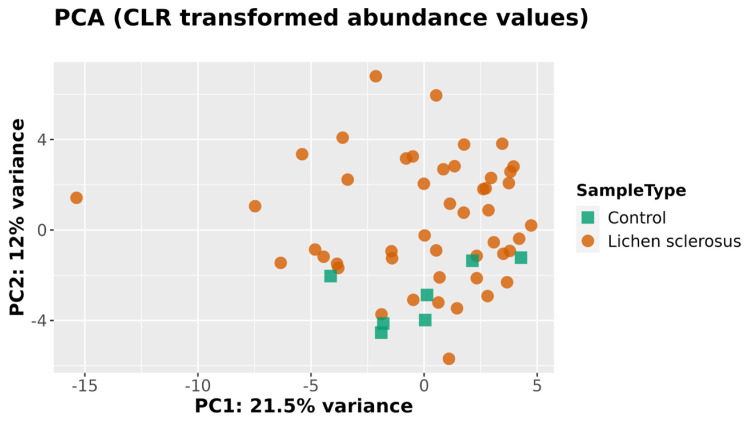
PCA clustering of samples after normalizing and transforming the 64 genera remaining after filtering for prevalence and frequency.

Centered log-ratio transformed abundance data for the 64 bacterial genera were clustered using PCA (Figure 2), with the healthy controls below 0 along the PC2 axis and the LSc samples scattered along the same axis.

**Figure 3:**
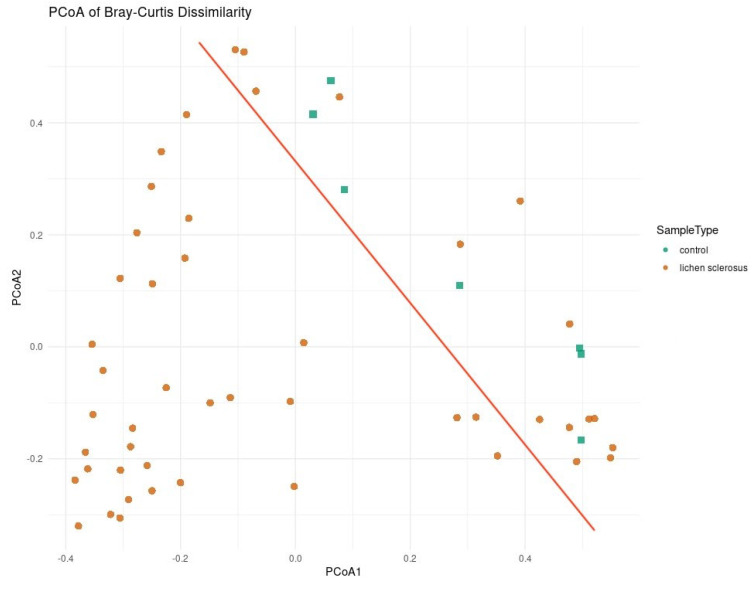
PCoA clustering of genital microbiome samples from healthy controls and women with LSc, including only the bacterial genera found to be significant by at least two methods of differential regression analysis.

**Figure 4:**
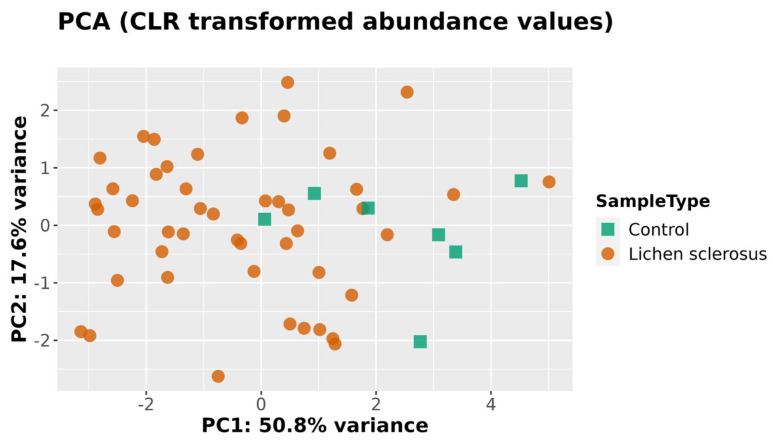
PCA clustering of genital microbiome samples from healthy controls and women with LSc, including only the bacterial genera found to be significant by at least two methods of differential regression analysis.

**Figure 5:**
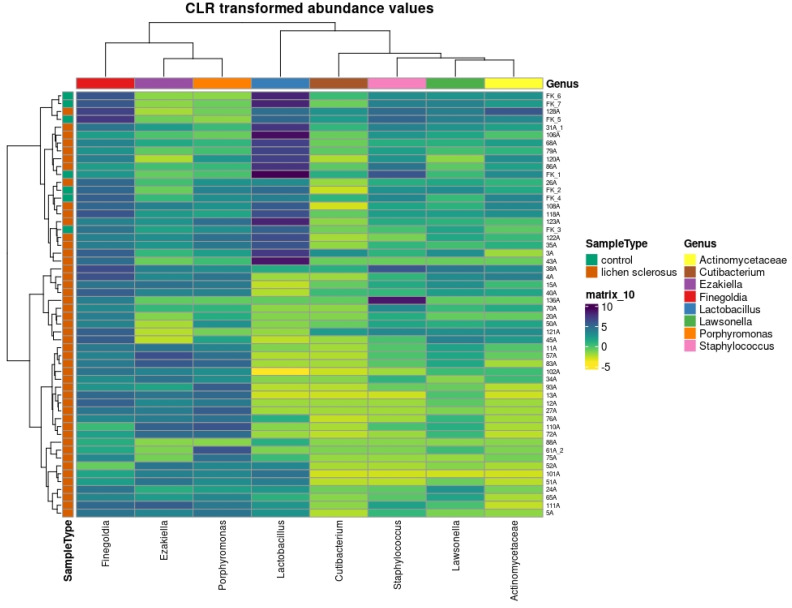
Heatmap depicting clustering of the bacterial genera found to be significant by at least two methods for differential regression analysis, in genital microbiome samples from healthy controls and women with LSc.

When analyzing the beta diversity with only the eight taxa found to be significant using at least two methods of regression analysis, clustering was found using PCoA (Figure 3), PCA (Figure 4), and a heatmap (Figure 5). In all three methods, the healthy controls were partially separated from the women with LSc.

## 5. Discussion

In this study, we observed a lower abundance of *Lactobacillus* in the anogenital microbiome of LSc patients compared to healthy controls, aligning with the findings of Liu et al. [15] but contrasting with those of Brunner et al. [14] and Nygaard et al. [17], who reported either no difference or *Lactobacillus* dominance in both groups. Notably, a subset of individual samples in our LSc group exhibited *Lactobacillus* levels comparable to the controls, suggesting that this variation may be due to interindividual differences or the small number of controls limiting interpretability.

*Lactobacillus* plays a protective role in the genital microbiome by enhancing epithelial adherence and inhibiting pathogenic colonization [20].

We found no significant difference in *Streptococcus* abundance between the two groups, consistent with findings of Brunner et al. [14] and Liu et al. [15], but in contrast to those of Nygaard et al. [17], who reported increased *Streptococcus* levels in LSc patients.

Our data also demonstrate increased abundance of *Ezakiella* in LSc patients, consistent with the findings of Liu et al. [15]. Notably, Taylor et al. [16] reported that *Ezakiella* abundance showed opposite associations with symptoms depending on anatomical site (vaginal versus vulvar), highlighting the need for further investigation into site-specific microbial dynamics.

A subgroup of samples was clustered based on similarities in *Lactobacillus* and *Ezakiella* abundance, suggesting the presence of distinct microbial subtypes within the LSc population, which may correlate with clinical heterogeneity.

We also observed a reduction in *Staphylococcus* abundance among LSc patients. This contrasts with the findings of Taylor et al. [16] who reported *Staphylococcus* as a dominant genus in the vulva, and Pagan et al. [12], who identified it in healthy vulvar skin. Liu et al. [15], by contrast, reported no significant difference, indicating that variability in *Staphylococcus* abundance may be influenced by hormonal status or other host-related factors.

We observed increased abundance of *Porphyromonas* in our LSc cohort, aligning with the findings of Chattopadhyay et al. [21] in girls, but contrasting with those of Liu et al. [15], who found no such increase in postmenopausal women, again suggesting a possible influence of age or hormonal status on microbial composition.

Our study also revealed reduced abundance of several lesser-studied genera, including *Finegoldia*, *Cutibacterium*, and *Lawsonella*. While Liu et al. [15] reported increased *Finegoldia* in LSc, we observed the opposite. Notably, *Finegoldia* magna is known to activate neutrophils and has been implicated in inflammatory skin conditions, like rosacea and eczema [22,23,24,25]. *Cutibacterium* and *Lawsonella* have also been associated with skin disorders, including psoriasis and alopecia [22,26]. However, given the relatively high FDR threshold employed, these findings should be interpreted with caution.

We hypothesize that the genera found to differ between LSc patients and healthy controls may play a functional role in disease pathogenesis. For example, the increased presence of anaerobic genera, such as *Ezakiella* and *Porphyromonas* in LSc samples, could suggest a shift toward a pro-inflammatory or tissue-degrading microbial environment. These organisms may contribute to local immune activation or disruption of the epithelial barrier, although their precise role remains unclear. Conversely, the decreased abundance of commensal genera typically associated with healthy skin, including *Cutibacterium*, *Staphylococcus*, and *Finegoldia*, may reflect a loss of microbial homeostasis or suppression of protective species. It is also possible that host factors, such as immune status, hormonal changes, or epithelial alterations, in LSc patients create a niche that favors these microbial shifts. Differences between our findings and those of previous studies may be explained by variation in sample site, patient age, hormonal status, or sequencing methods. Overall, these observations suggest that specific microbial profiles may either contribute to or result from the pathophysiological processes underlying LSc.

Beta diversity analysis in our cohort revealed significant differences between LSc patients and healthy controls. Similar findings have been reported [15,17], though these appeared to vary by anatomical site. In contrast, others have reported mixed results, with outcomes dependent on the clustering methods used and subgroup analyses performed [12,14].

The microbial alterations observed in LSc patients may reflect a multifactorial interplay between host and environmental factors rather than disease status alone. One plausible hypothesis for the reduced abundance of *Lactobacillus* in a subset of LSc patients is that local immune dysregulation and epithelial barrier dysfunction may impair colonization by protective species, while favoring the expansion of anaerobic taxa, such as *Ezakiella* and *Porphyromonas*. This microbial shift could both reflect and reinforce inflammation. Additionally, hormonal influences, particularly estrogen deficiency in postmenopausal women, could contribute to the observed microbial composition, consistent with prior evidence linking menopause to reduced *Lactobacillus* and increased microbial diversity. Age-related mucosal atrophy may further modulate microbial niches. The high interindividual variability observed, including *Lactobacillus*-dominant profiles within the LSc group, may therefore stem from differences in hormonal status, comorbidities (e.g., diabetes, atopy), or medication use, which were not captured in our metadata. This underscores the need for future studies to integrate clinical phenotyping with microbiome profiling in order to better contextualize microbial shifts within patient-specific backgrounds.

### Study Limitations

Despite the relatively large LSc cohort, the small number of healthy controls (n = 7) may limit statistical power and generalizability. A relatively high FDR threshold of 25% was used to minimize false negatives, but this increases the risk of false positives. Additionally, filtering criteria may have excluded rare but potentially important taxa.

The absence of clinical metadata (such as body mass index, hormonal status, menstrual cycle phase, hygiene practices, sexual activity, and medication use) limits interpretation of the observed microbial associations. Furthermore, the use of partial 16S rRNA gene sequencing restricts taxonomic resolution to the genus level and does not account for absolute bacterial load. More comprehensive approaches, such as shotgun metagenomic sequencing could offer improved taxonomic and functional resolution [27].

As a cross-sectional study, our design precludes assessment of temporal changes and causal relationships. Finally, all participants were recruited from a single center in Sweden, limiting generalizability to other populations. Future multi-center, longitudinal studies with richer clinical metadata and higher-resolution sequencing are needed to validate and extend these findings.

Although this was a single-center study, clinical metadata, such as BMI, hormonal status, hygiene practices, medication use, menstrual cycle phase, and sexual activity were not available at the time of analysis. While technically feasible to collect retrospectively, ethical and data protection limitations at our institution restricted access to certain sensitive personal health information without explicit participant consent for secondary analysis. Moreover, uniformity and completeness of metadata in hospital systems, particularly regarding lifestyle or hormonal variables, is often inconsistent in retrospective datasets. Recognizing the value of these variables, future studies from our group will integrate structured clinical data collection into the study design to enable more detailed microbiome–host interaction analyses.

## 6. Conclusions

This study identified notable differences in the anogenital microbiome of women with LSc compared to healthy controls. Specifically, the women with LSc exhibited reduced abundances of *Lactobacillus*, *Finegoldia*, *Lawsonella*, *Staphylococcus*, *Cutibacterium*, and an unclassified genus within *Actinomycetaceae*, alongside increased abundances of *Ezakiella* and *Porphyromonas*.

Despite growing interest in this area, previous studies have been limited by inconsistent findings and small sample sizes. Our results reinforce the need for larger, well-controlled investigations, with particular attention to variation by sex, age, disease location, and activity.

Microbiome research offers a promising avenue for improving our understanding of LSc pathogenesis. A clearer characterization of microbial patterns may eventually support the development of diagnostic, prognostic, and therapeutic tools for this chronic and often distressing genital dermatosis.

## Figures and Tables

**Table 1:** The abundance of bacterial genera in the anogenital region in women with LSc compared to that of healthy controls shown in log2 scale after differential analysis using DeSeq2, MaAslin CLR or limma. Adjusted *p* < 0.25.

	DeSeq2	MaAslin CLR	Limma
*Lactobacillus*	−3.47	−4.66	−7.16
*Finegoldia*	−1.85	−1.43	−2.36
*Lawsonella*	−1.62	−1.55	−2.49
*Satphylococcus*	Not significant	−2.79	−3.52
*Cutibacterium*	Not significant	−1.29	−3.12
Unknown Genus Belonging to *Actinomycetaceae*	Not significant	−2.28	−3.58
*Porphyromonas*	2.07	2.06	Not significant
*Ezekiella*	4.18	2.42	Not significant

## Data Availability

Data and materials can be assessed by contacting one of the authors.
